# Modafinil Alters Intrinsic Functional Connectivity of the Right Posterior Insula: A Pharmacological Resting State fMRI Study

**DOI:** 10.1371/journal.pone.0107145

**Published:** 2014-09-19

**Authors:** Nicoletta Cera, Armando Tartaro, Stefano L. Sensi

**Affiliations:** 1 Department of Neuroscience and Imaging, University “G. d'Annunzio” Chieti-Pescara, Chieti, Italy; 2 Molecular Neurology Unit, Center of Excellence on Aging, University “G. d'Annunzio”, Chieti-Pescara, Chieti, Italy; 3 Departments of Neurology and Pharmacology, Institute for Memory Impairments and Neurological Disorders, University of California-Irvine, Irvine, CA, United States of America; VU University Medical Center, Netherlands

## Abstract

**Background:**

Modafinil is employed for the treatment of narcolepsy and has also been, off-label, used to treat cognitive dysfunction in neuropsychiatric disorders. In a previous study, we have reported that single dose administration of modafinil in healthy young subjects enhances fluid reasoning and affects resting state activity in the Fronto Parietal Control (FPC) and Dorsal Attention (DAN) networks. No changes were found in the Salience Network (SN), a surprising result as the network is involved in the modulation of emotional and fluid reasoning. The insula is crucial hub of the SN and functionally divided in anterior and posterior subregions.

**Methodology:**

Using a seed-based approach, we have now analyzed effects of modafinil on the functional connectivity (FC) of insular subregions.

**Principal Findings:**

Analysis of FC with resting state fMRI (rs-FMRI) revealed increased FC between the right posterior insula and the putamen, the superior frontal gyrus and the anterior cingulate cortex in the modafinil-treated group.

**Conclusions:**

Modafinil is considered a putative cognitive enhancer. The rs-fMRI modifications that we have found are consistent with the drug cognitive enhancing properties and indicate subregional targets of action.

**Trial Registration:**

ClinicalTrials.gov NCT01684306

## Introduction

Modafinil is a compound employed in the treatment of sleep disorders, and, off-label, also used for the treatment of cognitive deficits in schizophrenia, Attention Deficit/Hyperactivity Disorder (ADHD), and mood disorders [1]–[5]. Previous preclinical and human studies have indicated that modafinil modulates neurotransmission in several brain regions including the hypothalamus, hippocampus, basal ganglia and prefrontal regions. The compound acts on orexin, monoamines and dopamine as well as on glutamate, and gamma-aminobutyric acid [6]–[8]. Recent studies have indicated that modafinil positively modulates attention, memory, and executive functions [9], [10]. Modafinil can be considered a cognitive enhancer that, compared to amphetamine-like psychostimulants, may have lower risks of inducing addiction [11]–[13].

In a previous study, we have reported that the administration of a single dose (100 mg) of modafinil affects the brain resting state network (RSN) activity in healthy young individuals [14]. The study showed that, of six selected RSNs [the Default Mode Network, the Salience Network (SN), the Fronto Parietal Control network (FPC; lateralized in both hemispheres), the Sensory Motor Network, the Exstrastriate Visual System and the Dorsal Attention Network (DAN)], functional connectivity (FC) effects were found only in the FPC and DAN. No statistically significant modifications were observed in the SN.

The SN is composed of three nodes. The SN includes the bilateral insular cortices and dorsal anterior cingulate cortex as well as subcortical structures like the amygdala, the substantia nigra/ventral tegmental area, and the thalamus [15]. The SN plays an important role in controlling attention toward biologically-relevant and cognitively-relevant stimuli present in the environment [15], [16], an overall function that helps to guide behavior.

Several imaging studies have shown that the insula and cingulate cortex are simultaneously activated upon cognitive tasks [17]. The insula is indeed considered a critical hub that mediates the information flow within the SN [15], [16]. The region interacts with limbic, somatosensory, and motor regions and is also crucial in the coordination of sensorimotor, visceral, and interoceptive processing as well as in controlling homeostatic/allostatic functions like self-awareness and empathy [18]–[20]. The insula is also critical in controlling motivation, a process that is partly mediated by the activation of the orexin receptor, a preferential pharmacological target for modafinil [21]. In summary, functioning of the insula is important in the response to salient environmental stimuli as the region promotes a spatio-temporal integration that is needed for cognitive and emotional elaborations [16].

Several studies have shown functional sub-differentiations within the insular cortex when considering the activity of the anterior versus posterior portions of the region. The anterior part appears to be mainly involved in cognitive and socio-cognitive functions such as empathy processing, emotional salience detection, and attentional control [15]; [22]–[24]. The posterior part modulates sensorimotor tasks [25]. These different functions are matched by distinct connectivity patterns between the two subregions and other brain areas. The anterior insula is functionally connected with the Anterior Cingulate Cortex (ACC) whereas the posterior insula appears to be mainly connected with the somatosensory and middle cingulate cortices [16], [17], [26].

In a previous study, we have observed that healthy young individuals, after modafinil administration, showed improvement in fluid reasoning as measured with Advanced Progressive Matrices (APM) [14], [27]. Previous imaging studies have indicated that SN nodes, i.e.: bilateral insulae and the cingulate cortex are involved in tasks set to stimulate fluid reasoning [28], [29] but in our study were unable to detect significant modafinil effects on SN activation [14]. In the past decade, resting state fMRI (rs-fMRI) has emerged as valuable tool for the study of neural activity when the brain is at rest and not involved in task completion. Compared to task-related fMRI, rs-fMRI offers the advantage of allowing the investigation of simultaneous and coordinated activity of multiple and well-defined brain networks. This approach also reduces confounding factors like the inter-individual variability in task compliance and/or performance that can occur upon fMRI acquisition [14]. rs-fMRI has been successfully employed to evaluate FC modifications [30]. FC is studied with MRI through the analysis of simultaneous variations of BOLD (Blood-Oxygen-Level-Dependent) signals occurring in distinct brain regions [24], [31], [32]. FC can be studied at rest by evaluating spontaneous low frequency fluctuations of BOLD signals in different brain voxels. The process allows the identification of temporally-related patterns of activity across brain regions that are involved in similar or related functions [33].

Given the subregional differentiation of the insular cortex and its role in the SN, we have decided to re-analyze our fMRI data with the aim of exploring modafinil-induced subregional patterns of FC that is occurring within the insulae as these insular subregions are central for the beneficial cognitive effects we have observed after a single dose exposure to the drug [14].

To that aim, fMRI acquisitions obtained in the previous study were now analyzed with a seed-based approach that was focused only on activity occurring in the left and right insulae divided in anterior and posterior subregions.

## Materials and Methods

### Ethics Statement

The protocol for this trial and supporting CONSORT checklist are available as supporting information; see Checklist S1 and Protocol S1.

The study was approved by the ethics committee of University of Chieti (PROT 2008/09 COET on 14/10/2009) and conducted in accordance with the Helsinki Declaration.

The study design was explained in detail and written informed consent was obtained from all participants involved in our study. Recruitment was performed throughout February 2011, drug/placebo administration and fMRI acquisitions started on March 2011, went on until January 2102, and the study was completed with the last fMRI session in January 2012. After securing financial coverage for costs related to the analysis of the study, the trial was registered on 10/09/2012 (ClinicalTrials.gov NCT01684306 http://clinicaltrials.gov/ct2/show/NCT01684306). After obtaining registration, the double blind study was opened and analyzed, rs-fMRI data were investigated by means of independent component analysis (ICA) leading to a first publication of the dataset[14]. The protocol is the same as the previous study [14] with no deviations made. The authors confirm that all ongoing and related trials for these drug/interventions are registered.

### Study group, design, and rs-fMRI acquisition

**Figure 1:**
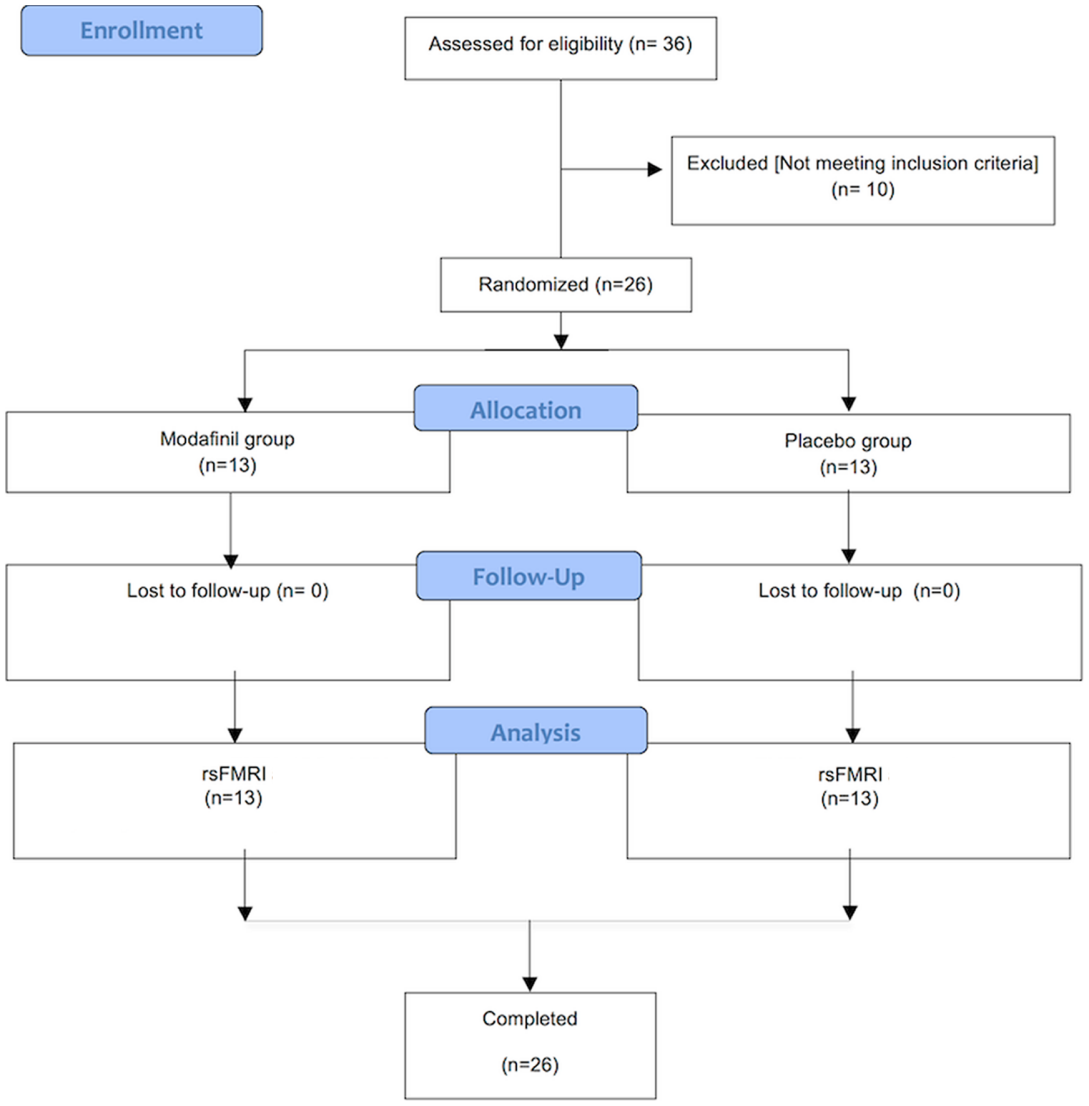
CONSORT Diagram. Flow diagram graphically describes the design of the study: enrollment, intervention, follow-up and data analysis.

The study group, experimental design, and rs-fMRI data acquisition are described in the original study [14; Figure 1]. Randomization of study subjects was obtained by means of random number generator. In our study, in line with similar pharmacological-fMRI studies, and also considering that we did not have preliminary data that could be used to, a priori, estimate the optimal sample size, we have evaluated two groups of 13 subjects that is nowadays an accepted size in these kind of studies.

### MRI/fMRI data analysis

MRI and fMRI data were analyzed with the Brain Voyager QX 2.3 software (Brain Innovation, Maastricht, The Netherlands). Preprocessing of functional data was performed by sequentially applying slice scan time correction, three-dimensional motion correction, and removal of linear trends from voxel time series. Preprocessed functional volumes were then co-registered with the corresponding structural dataset. Both structural and functional volumes were then transformed into the Talairach space [34] using a piecewise affine and continuous transformation. Functional volumes were re-sampled at a voxel size of 3×3×3 mm^3^. A spatial smoothing with a Gaussian kernel of 6.0 mm full-width half-maximum was applied to functional images corresponding to two voxels in the re-sampled data to account for intersubject variability while maintaining a relatively high spatial resolution.

### FC analysis

Previous studies have identified different subregions in the insular cortices in both humans and non-human primates [35]. These subregions appear to show different connectivity patterns with remaining brain regions. To examine FC patterns in the insular cortices, four ROIs were selected for each subject, before and after drug/placebo administrations, within the anterior portion of the left hemisphere (AIlh) or right hemisphere (AIrh) and posterior portions of the left (PIlh) or right hemisphere (PIrh). Regions of interest (ROIs) were determined using Talairach coordinates. Talairach coordinates are defined on the basis of two brain structures: the anterior and posterior commissures. Distances in Talairach coordinates are measured from the anterior commissure that is, by convention, intended as the origin [34]. The y-axis represents the anterior

->posterior direction, the x-axis represents the left->right, and the z-axis is depicts the dorsal->ventral direction (Table 1, [34]). Each ROI was created by means of TalCoord2VOI plug-in with a radius of 2,5 mm to avoid White Matter (WM) inclusions.

**Table 1:** Between-group comparison for right posterior insula for the contrast T1>T0.

Cluster	BA	Hemisphere	X	Y	Z	Nr. Voxels
SFG	6	R	55.3	−4.1	23.1	60
MTG	22	R	46.9	−407.	15.8	1053
Putamen		R	27.8	9	1.1	197
Precuneus	29/30	R	18.1	−55.9	28.5	159
ACC	24	R	10.2	−20	31.5	317
SFG	6	R	0.6	−13.9	55.4	2643
dorsal PCC	31	R	2.7	−20	37.9	137
ACC	24	L	−12.7	−2.9	31.7	855
Putamen		L	−22.8	1	−4.7	118
Posterior Insula/temporal pole	13/38/34	L	−45.4	−4	−0.1	2836
Anterior prefrontal cortex	10	L	−33.2	26.9	20.2	68

Table indicates brain regions showing significant differences when considering T1 (drug >placebo)>T0 (drug>placebo) for the right Posterior Insula (PIrh). Brain regions are listed with the mean Talairach coordinates (x: left-right; y: anterior-posterior; z: dorsal ventral) and the corresponding number of voxels.

Abbreviations: BA: Brodmann's area; L: left; R: right. PCC: Posterior Cingulate Cortex; MTG: Middle Temporal gyrus; ACC: Anterior Cingulate cortex; SFG: Superior Frontal gyrus.

Whole brain seed-based connectivity maps, related to the selected ROIs (see above), were created for all subjects. We then calculated correlations between ROI time-courses (i.e.: the time-course in each of the insula subregions) and all the time-courses of the brain voxels [36]. BOLD time-courses were extracted from each ROI by obtaining an average value for each voxel of the ROI modeled for each single subject. To reveal FC patterns that were consistent for the groups along with the T0 and T1 time points in relation to each insular subregions, we proceeded in the following way: after applying the Fisher's r-to-z transformation to each correlation map, random-effect analysis was independently performed for each of the two study groups and the two acquisition time points.

FC maps were computed according to Margulies et al. [37]. Nuisance covariates were included in the analyses to reduce effects of physiological processes such as fluctuations related to cardiac and respiratory cycles [38], [39] or to motion. To this aim, we included eight additional covariates that modeled nuisance signals sampled from WM and Cerebro-Spinal Fluid (CSF) [40], as well as from six motion parameters (3 rotations and 3 translations as saved by the 3D motion correction). We derived WM/CSF nuisance signals averaging voxel time courses in each subject whole brain WM/CSF masks. These masks were generated by the segmentation process of each subject brain by means of Brain voyager QX. All seed-based predictors were z-normalized and analyses repeated with each insular subdivision inserted in a separate regression model.

### Statistical analysis

For each seed ROI, subject, and condition (drug vs. placebo), and time (T0 vs. T1), a FC map was computed on a voxel-wise basis. For each subject, the general linear model (GLM) [41] for multiple regression analysis produced four ROI-based t-maps. To assess group differences between T0 and T1, four different voxel-wise mixed model Analyses of Variances were performed by means of the ANOVA tool of Brain Voyager QX set with one between-group factor (drug vs. placebo) and a repeated measure factor (T1 vs. T0). To control for absence of between-group differences, a between-group comparison was performed at T0. To assess effect of drug/placebo network FC of each ROI, we performed the contrast T1 (drug>placebo)> T0 (drug>placebo). Statistical significance was assessed by setting a threshold that was corrected by the False Discovery Rate (FDR) [q<0.02 corresponding to t>3.93 and p<0.001 at voxel level; 42]. To avoid circularity effects, statistical analyses were performed in accordance with what indicated by Kriegerskorte and colleagues [43].

## Results

**Figure 2:**
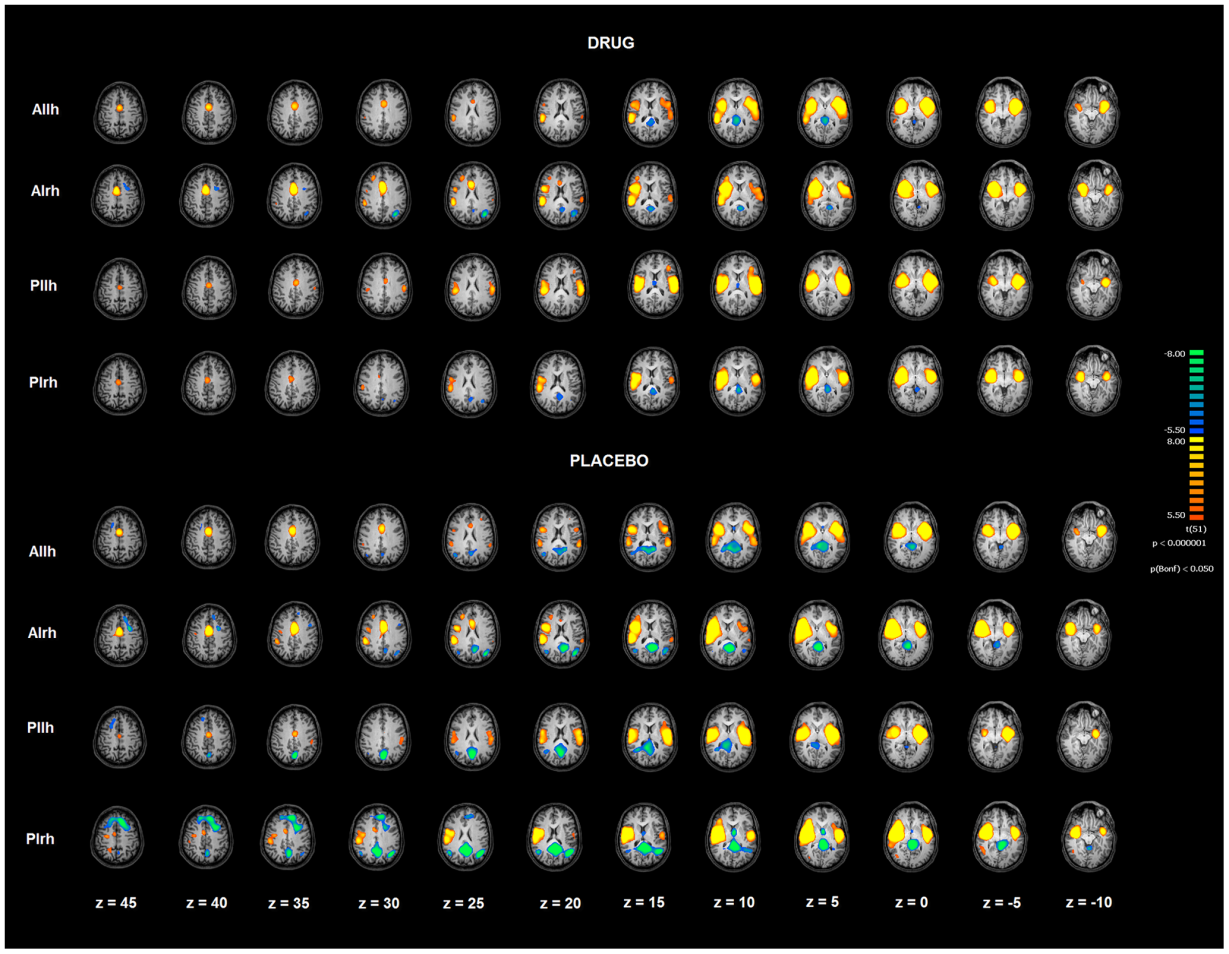
Insula functional connectivity patterns before drug/placebo treatment. Image depicts functional connectivity patterns of the four subregions of bilateral insulae as assessed with rs-fMRI. Maps are overimposed on a Talairach atlas and in radiological convention with a statistical significance set at p<0.05 Bonferroni corrected. AIlh = anterior insula left hemisphere; AIrh  = anterior insula right hemphere; Pilh = posterior insula left hemisphere; PIrh =  posterior insula right hemisphere.

**Figure 3:**
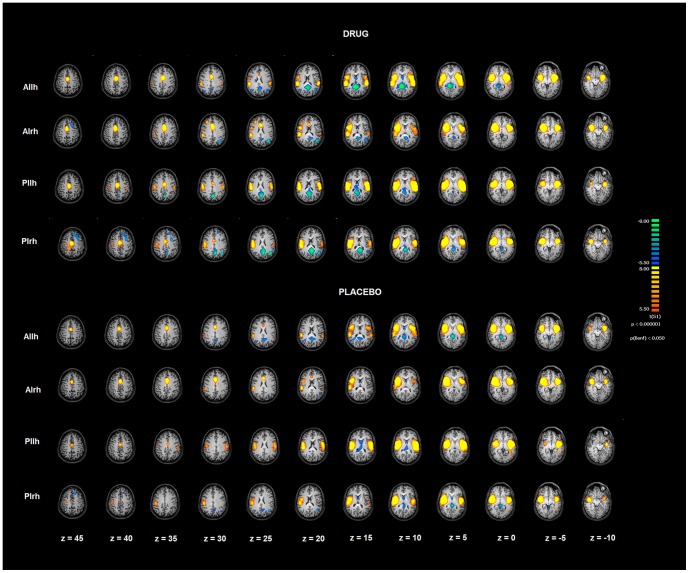
Between-group comparison of the right posterior insula pattern before and after drug/placebo treatment. Image depicts the map obtained after contrast T1 (drug>placebo) >T0 (drug>placebo) for the right posterior insula (PIrh). The map is overimposed on a Talairach atlas and in radiological convention (p<0.02 FDR corrected). Differences are assessed by means of a mixed model voxel wise ANOVA with a between-group factor (Drug vs Placebo) and a repeated measure factor (T0 vs. T1).

**Figure 4:**
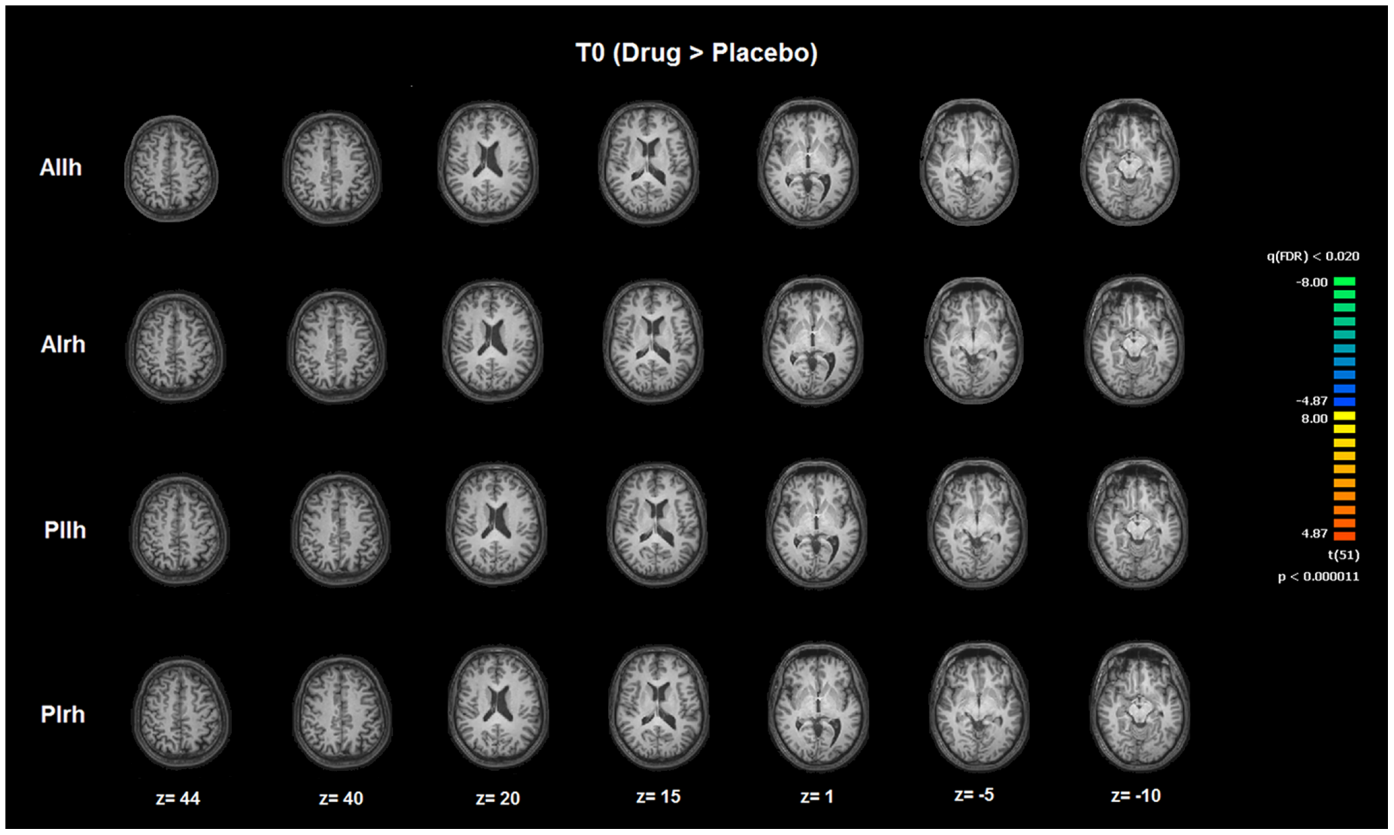
Between-group comparison of the four insula subregions before drug/placebo treatment. Image depicts maps obtained after contrast T0 (Drug>Placebo) for the four subregions of bilateral insulae as assessed with rs-fMRI. Maps are overimposed on a Talairach atlas and in radiological convention (p<0.02 FDR corrected). Differences are assessed by means of a mixed model voxel wise ANOVA with a between-group factor (Drug vs Placebo) and a repeated measure factor (T0 vs. T1) and specific contrast T0 (Drug>Placebo). AIlh = anterior insula left hemisphere; AIrh = anterior insula right hemphere; Pilh = posterior insula left hemisphere; PIrh = posterior insula right hemisphere.

Four patterns of ROI seed-based FC patterns were investigated in specific insular subdivisions. The analysis was performed on rs-FMRI data at T0 (before the administration of drug/placebo) and T1 (after the administration of drug/placebo). FC maps were calculated for each seed ROI [Anterior Insula left hemisphere (AIlh); Anterior Insula right hemisphere (AIrh); Posterior Insula left hemisphere (PIlh); Posterior Insula right hemisphere (PIrh)] and showed distinct patterns of connectivity in specific insular subregions. Resulting maps were corrected for multiple comparisons by means of Bonferroni correction with a threshold set at p<0.05 (Figure 2 and 3, Table 2). At T0, no differences were found in the two groups as far as FC of the four insular subregions with a p<0.02 FDR corrected (Figure 4).

**Table 2:** Insula subregional seed-based correlation.

Anterior insula - Left hemisphere	Seed: x = −38; y = 15; z = −2					
Cluster	BA	Hemisphere	X	Y	Z	Nr. Voxels
**T0 -Drug**						
Insula IFG pars opercularis and triangularis	13/44/45					
STG putamen	/22/41	R	43.2	−6.7	5.9	44739
Parahippocampus temporal pole amygdala		R	30.9	−28.2	−20.5	186
Retrosplenial PCC	29/31	L	−0.2	−39.1	9.1	8517
Ventral and dorsal ACC premotor cortex	24/32/6	R	11	0.2	40.8	10995
Insula IFG pars opercularis and triangularis	13/44/45					
STG putamen	/22/41	L	−41.3	0.5	2.6	49422
**T0 - Placebo**						
Insula IFG pars opercularis and triangularis	13/22/44					
STGputamen	/45/41	R	41.8	−1.9	6	42945
Retrosplenial PCC	23/29/31	R	4.5	−47.8	11.1	29163
Parahippocampus temporal pole amygdala	38	R	30.1	−24.5	−16.5	1091
MFG	8	R	20.7	16.7	43.3	685
Ventral and dorsal ACC premotor cortex	32/24/6	L	−0.1	1.2	39.4	13217
Insula IFG pars opercularis and triangularis	13/22/44					
STG putamen	/45/41	L	−42.5	−0.9	3.5	47833
Dorsolateral preforntal cortex	9	L	−31.5	36.2	24.4	284
**T1 - Drug**						
Insula IFG pars opercularis and triangularis						
STG putamen	44/47/13/40	R	43.9	−7.7	7.9	51049
Retrosplenial PCC	30/31/23	R	4.2	−47.2	13.5	28483
Parahippocampus temporal pole amygdala	38	R	30.5	−26.8	−19.1	334
Extra striate cortex	18	R	23.8	−78.5	−18.5	523
Cudate Nucleus		R	20.8	−12.9	25.5	496
Ventral and dorsal ACC premotor cortex	6/24/32	R	0	−2.7	40.6	16971
MFG	8	L	−13.1	31.9	40	65
Insula IFG pars opercularis and triangularis	13/45/44					
STGputamen	/41/42/22	L	−44.4	−6.6	4.1	57332
Augular and supramerginal gyrus	39/40	L	−42.1	−64	23.3	1865
**T1 - Placebo**						
Insula IFG pars opercularis and triangularis	44/45/13					
STG putamen	/22/40/41	R	43.1	−1.9	6.2	42772
Retrosplenial PCC	30/29/23/31	R	5.4	−46.9	13.2	18684
Ventral and dorsal ACC premotor cortex	6/24/32	R	0.5	2.7	38.8	11655
Insula IFG pars opercularis and triangularis	44/45/13					
STG putamen	/22/40/41	L	−42.2	−1.1	3.1	43055

Anterior insula - Right hemisphere	Seed: x = 37; y = 16; z = 3					
Cluster	BA	Hemisphere	X	Y	Z	Nr. Voxels
**T0 - Drug**						
Insula IFG pars opercularis and triangularis	13/22/44/45/4					
STGputamen thalamus	1	R	39.8	−2.0	6.6	70971
Ventral and dorsal ACC	32/24	R	3.2	0.3	40	23859
PCC	30	L	−2.9	−52.5	12.3	5600
SFG/MFG	6/8	L	−26.7	6.3	42.3	2167
Insula IFG pars opercularis and triangularis	13/22/44/45/4					
STGputamen thalamus	1	L	−43.4	−1.4	2	30606
	39	L	−36.6	−66.9	25.4	5522
**T0 - Placebo**						
Insula IFG pars opercularis and triangularis	13/22/44/45/4					
STGputamen thalamus	1	R	41.1	−3.5	7.9	84229
Angular and supramarginal gyrus	39/40	R	42.8	−65.7	18.7	1862
Ventral and dorsal ACC	32/24	R	3.9	−2.9	40.5	24955
Retrosplenial PCC	23/30/31	L	−5.2	−51.2	11.2	24438
SFG/MFG	6/8	L	−20.3	16.4	45	6172
Parahippocampus temporal pole amygdala		L	−27.8	−17.2	−22.3	1174
Insula IFG pars opercularis and triangularis	13/22/44/45/4					
STGputamen thalamus	1	L	−40.9	2	−0.3	23190
Angular and Supramarginal gyrus	39/40	L	−40.6	−66.9	20.1	5946
SFG premotor cortex	6	L	−44.2	10.2	33.6	537
STG	22/42	L	−60.1	−32.7	16.3	1413
**T1 - Drug**						
Insula IFG pars opercularis and triangularis	13/22/44/45/4					
STGputamen thalamus	1	R	39.5	−0.9	7.7	71046
Ventral and dorsal ACC	32/24	R	1.7	2.6	38.0	24485
Retrosplenial PCC	23/30/31	L	−5.4	−52.2	14.7	7612
SFG premotor cortex	6	L	−21.9	16.8	44.7	2611
Insula IFG pars opercularis and triangularis	13/22/44/45/4					
STGputamen thalamus	1	L	−42	−1.5	1.7	31194
Angular and supramarginal gyrus	39/40	L	−40.5	−64.2	23.2	6336
**T1 - Placebo**						
Insula IFG pars opercularis and triangularis	13/22/44/45/4					
STGputamen thalamus	1	R	40	−2	5.3	59401
Ventral and dorsal ACC	32/24	R	1.4	7.4	35.5	16035
Insula IFG pars opercularis and triangularis	13/22/44/45/4					
STGputamen thalamus	1	L	−38.0	3.7	−0.7	23429
Angular and Supramarginal gyrus	39/40	L	−41.7	−63.4	20.6	825
STG	22/42	L	−59.4	−33.5	18.5	382

Posterior insula – Left hemisphere	seed: x = −42; y = −9; z = 4					
Cluster	BA	Hemisphere	X	Y	Z	Nr. Voxels
**T0 – Drug**						
Insula IFG pars opercularis and triangularis	13/22/44					
STG putamen	/45/41	R	43.9	−8.9	8.8	44235
ACC	24	L	−0.5	−6.9	37.5	4060
	13/22/44/					
Insula IFG pars opercularis and triangularis pre and post central gyrus STG putamen	45/41/2/4/3/6	L	−44.7	−8	6.7	62929
**T0 – Placebo**						
Insula IFG pars opercularis and triangularis	13/22/					
STG putamen	44/45/41	R	43.7	−7.6	9.3	36180
PCC SPL	23/30/31/7	R	6	−52.4	18.9	33283
MFG	8	R	20	23.8	44.5	2378
ACC	24	L	−1.4	−8.2	38.2	3288
	13/22/44/					
Insula IFG pars opercularis and triangularis pre and post central gyrus STG putamen	45/41/2/4/3/6	L	−43.6	−8.7	7.7	58956
**T1 – Drug**						
Insula IFG pars opercularis and triangularis pre and post central gyrus STGputamen	13/22/44/45/41/2/3/3/6	R	44.7	−10.2	11	54610
PCC SPL	23/30/31/7	L	−0.4	−49.8	20.2	22724
ACC	24	R	0.1	−11.5	42.3	14520
Insula IFG pars opercularis and triangularis pre and post central gyrus STGputamen	13/22/44/45/41/2/4/3/6	L	−46.5	−9.4	8.2	64141
Angular gyrus	39	L	−43.2	−63.7	22.3	64
**T1 – Placebo**						
Insula IFG pars opercularis and triangularis	13/22/44/					
STG putamen	45/41	R	45.2	−7.9	11.2	42828
ACC	24	R	0.5	−8.9	42.1	5533
Insula IFG pars opercularis and triangularis pre and post central gyrus STGputamen	13/22/44/45/41/2/4/3/6	L	−45.5	−11.1	8.3	59758

Posterior insula - Right hemisphere	seed: x = 39; y = −9; z = 9					
Cluster	BA	Hemisphere	X	Y	Z	Nr. Voxels
**T0 - Drug**						
Insula IFG pars opercularis and triangularis	13/22/44/					
STG putamen	45/41	R	43.4	−7.6	6.3	55307
SPL	7	R	18.3	−48.1	51.8	122
SFG ventral and dorsal ACC	6/32/24	R	4.8	−10.1	44.6	6387
PCC	23/30/31	L	−3.9	−44.2	12.2	8458
Insula IFG pars opercularis and triangularis	13/22/44/					
STG putamen	45/41	L	−44.9	−3.7	1.2	26362
Angular gyrus	39	L	−38.7	−65.1	25.5	728
**T0 - Placebo**						
Insula IFG pars opercularis and triangularis	13/22/24 44/					
ACC STG putamen	45/41	R	40.8	−7.6	10.6	91610
PCC angular gyrus	23/30/31	L	−11.1	−46.8	15.4	67270
Prefrontal cortex	8/10	L	−9.7	25.7	40.3	37524
Angular gyrus	39	R	−44.2	−59.6	22.6	3245
Insula IFG pars opercularis and triangularis	13/22/44/					
STG putamen	45/41	L	−46.8	−6.5	0.8	25923
SPL	7	R	19.7	−52.7	4.8	1229
**T1 - Drug**						
Insula IFG pars opercularis and triangularis	13/22/44/					
STG putamen	45/41	R	43.4	−9.1	10	65731
ACC SFG pre and postcentral gyrus	24/6/1/2/3	R	6.8	−19.2	46.2	25291
PCC	23/30/31	L	−3.2	−51.4	18	24282
Dorsolateral prefrontal cortex	9	L	−20.6	22.1	40.9	10736
Insula IFG pars opercularis and triangularis	13/22/44/					
STG putamen	45/41	L	−47.6	−6.7	4.1	29616
Angular gyrus	39	L	−42	−63.7	24.1	5383
Angular gyrus	39	R	45.4	−62.6	23.2	254
**T1 - Placebo**						
Insula IFG pars opercularis and triangularis	13/22/44/45/41/6/3/4/					
STGputamen pre and postcentral gyrus	2	R	43.9	−8.9	9.7	58139
SPL	7	R	20.9	−47.1	51.9	1826
Caudate nucleus		R	13.7	22.2	11.6	436
SFG ACC	24/6	R	4.8	−15.4	47.8	1741
Precuneus	29	L	−5.9	−59.8	26	2072
PCC	23/29	L	−2.5	−36.1	4.2	5523
MFG	8	L	−12	26.6	46.4	1861
SPL	7	L	−24.1	−41.7	54.9	321
Insula IFG pars opercularis and triangularis	13/22/44/45/					
STG putamen	41	L	−46.8	−8.2	1.9	21263
Angular gyrus	39	L	−41	−61.9	23.5	2094

Table indicates brain regions showing seed-based functional connectivity [with significance level set at P<0.05 (Bonferroni corrected)] for the left anterior (AIlh) right anterior (AIRh) left posterior (PIlh) and right posterior (PIrh) at T0 or T1 respectively. Brain regions are listed according to mean Talairach coordinates (x: left-right; y: anterior-posterior; z: dorsal ventral) and corresponding number of voxels.

Abbreviations: BA: Brodmann's area; L: left; R: right. PCC: Posterior Cingulate Cortex; MFG: Middle frontal gyrus; STG:Superior Temporal gyrus; IFG: Inferior Frontal gyrus; ACC: Anterior Cingulate cortex; SPL: Superior Parietal Lobe; SFG: Superior Frontal gyrus.

Baseline analysis at T0 confirmed known distinct patterns of connectivity of the four insular subregions. AIlh and AIrh showed significant FC with the ipsilateral inferior frontal gyrus (IFG), the posterior insular cortex, the anterior middle cingulate cortex (aMCC), the ACC, the superior frontal gyrus (SFG), the contralateral anterior and posterior insula. For PIlh and PIrh, FC was found with the putamen bilaterally, the dorsal ACC and the precentral and postcentral gyri as well as with the contralateral anterior and posterior insulae.

At T1, both study groups showed FC maps for AIlh and AIrh that were largely similar to the ones found at T0. AIlh and AIrh showed significant FC with the above listed T0 regions with the addition of the superior temporal gyrus (STG) and the dorsolateral prefrontal cortex (dlPFC). At T1, maps for PIlh and PIrh indicated FC with the contralateral posterior/middle insula, the putamen, the parahyppocampus, the MCC, the precentral/postcentral gyri, and the superior parietal lobule (SPL).

We then performed a comparison of T1 (drug>placebo) versus T0 (drug>placebo) for each seed ROI maps setting significance levels at p<0.02 FDR corrected. AIlh, AIrh and PIlh showed no significant differences. The only significant difference was observed for PIrh. The regions showed, bilaterally, a significant FC increase in the SFG, putamen, and dorsal ACC. A lateralized effect was found in the right hemisphere with significant differences in the middle temporal gyrus whereas significant between-group differences were observed, at T1>T0, for the left posterior insula and left temporal pole up to the parahippocampal region (Figure 3, Table 1).

## Discussion

**Figure 5:**
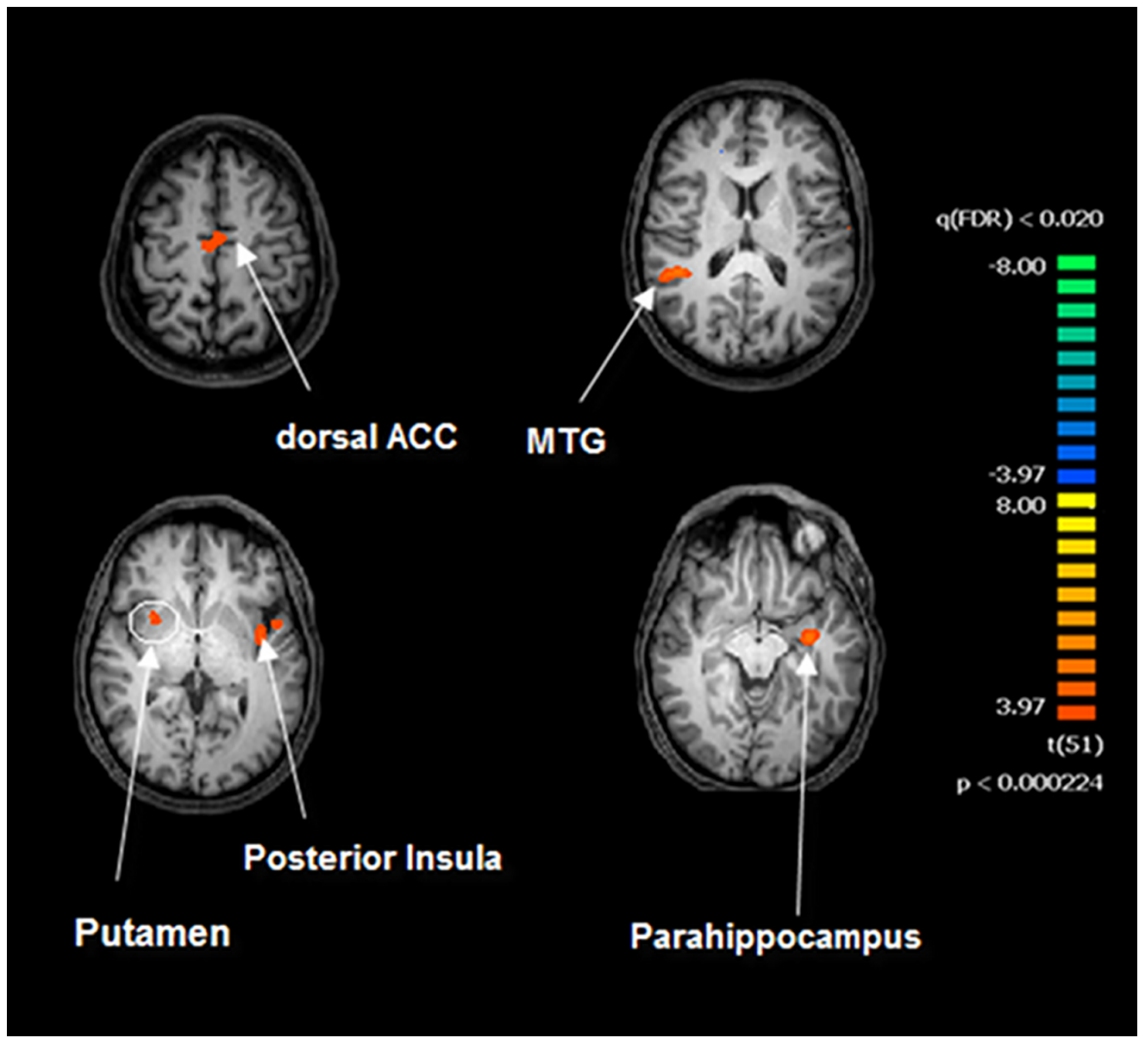
Insula functional connectivity patterns after drug/placebo treatment. Image depicts functional connectivity patterns of the four subregions of bilateral insulae as assessed with rs-fMRI. Maps are overimposed on a Talairach atlas and in radiological convention with a statistical significance of p<0.05 Bonferroni corrected. AIlh = anterior insula left hemisphere; AIrh = anterior insula right hemphere; Pilh = posterior insula left hemisphere; PIrh = posterior insula right hemisphere.

In the present study, we investigated subregional FC effects of modafinil in the insula. Posterior and anterior insular cortices showed differential functional behavior (Figure 2 and 5, Table 2).

In the modafinil group, after drug analysis revealed distinctive FC patterns within nodes of the right posterior insula. After modafinil administration, we found increased FC levels in the putamen, left parahippocampus, left posterior insula and MCC (Figure 3, Table 2). Our results indicate that the drug does not reorganize FC within sub-regions of the insula but strengthens the region overall connectivity, a phenomenon commonly observed in fMRI studies [44].

In a previous study, we have found that a single dose administration of modafinil does not modify SN activity [14]. However, previous findings indicate that the anterior insula, a major SN hub [15], is activated by modafinil in methamphetamine addicts who were undergoing a reversal learning task [13]. The anterior insula has also been shown to be involved in tasks focused on modulation of fluid reasoning [28], another cognitive function that we have found improved in modafinil treated subjects [14].

Seed-based analysis of functioning of insular subregions offered additional information that could help to reconcile these divergent results.

Our within-group results showed patterns of FC occurring between the anterior insula and frontal regions, the ACC, and the controlateral insula. The posterior insula showed FC with the putamen bilaterally, the dorsal ACC, and the precentral and postcentral gyri as well as with the contralateral anterior and posterior insulae. These FC results are in line with known anatomical connections [35].

Previous rs-fMRI studies have shown that the insula is involved in two distinct neural networks. The first network links the anterior insula to the ACC, the prefrontal and frontal cortex as well as to parietal and temporal regions. The second network links the posterior insular cortex to the middle cingulate, sensorimotor, premotor, and temporoparietal cortices. This posterior pattern is mainly involved in motor functions such as body orientation, monitoring of the environment and response selection [17], [18]; [45]–[47].

The two networks communicate with a posterior-anterior modality [18]. Thus, a model can be envisioned by which thalamocortical pathways send a representation of homeostatic information to the posterior insula, thereby generating distinct interoceptive feelings that are projecting onto anterior insula to help in promoting emotional evaluation.

The anterior portion of the insula appears to have a more defined role in the interplay between high cognitive and emotional functions. In addition, compared to the left insula, the right region has a more prominent role in the exploration of the environment and spatial orientation. In particular, the right insula plays a fundamental role in spatial self-orientation and awareness of limb movements in space [48]–[50].

While many studies have dissected the role of the anterior insula, fewer reports have analyzed functioning of the posterior insula [43].

The right posterior insula appears to be more involved in monitoring the external environment as well as in response selection and action preparation. Several studies indicate that this region is an area of convergence for processing multimodal exteroceptive, interoceptive, vestibular, and auditory stimuli.

In our study, it is conceivable that the drug-dependent increase of FC that we observed between the right insula and putamen can promote enhanced monitoring of internal states aimed at motor planning. The overall nature of the insula-putamen connection lends some support to this hypothesis.

The putamen is a region involved in motivation and reward-related learning [51], the area is in fact important to manage motor actions aimed at obtaining reward [52], [53].

The putamen and posterior insula are part of a network that controls decision making processes and impulsivity [54]–[56]. The dorsal-posterior insula also represents a key region for time encoding [57].

The putamen activity helps in recognizing emotions and bodily states as well as motivation [58], [59] and is thought to be driven by increased FC in the MCC, an area involved in goal-directed behavior. The ACC, a region that is functionally and structurally connected to the dorsal striatum, is involved in action planning and motivation. The cingulate cortex is important for social behavior and, supporting this concept, ACC lesions have been shown to lead to akinetic mutism and apathy [60], [61].

The hippocampus and insula work together in visuospatial exploration [62]. The parahippocampal regions are also involved in reward processing through activation of the ventral striatum [63]–[66]. Thus, one can speculate that modafinil may act in the early stages of cognitive processing that are associated with action preparation as well as motivation to act. In that respect, the right posterior insula can sustain motivation by working in synergy with the ACC and putamen along with the intervention of parahippocampal regions that are responsible for long-term storage of reward-related memories. Preclinical studies have clarified the role of insular orexin receptors in modulating motivation and lend support to our hypothesis as modafinil is a strong orexin receptor agonist [21].

Modafinil-dependent modifications of the right posterior-insula network activity may explain the pro-cognitive effects of the drug. Further research is warrant to evaluate the selective role of the posterior insula as important target for cognitive enhancing drugs.

## Supporting Information

Checklist S1 **CONSORT 2010 checklist of information included in the modafinil randomised trial.**
(PDF)

Protocol S1 **Ethic committee approval of the protocol.**
(PDF)
